# Atypical Giant Lipoma in the Left Lower Abdomen: A Case Study

**DOI:** 10.7759/cureus.59930

**Published:** 2024-05-08

**Authors:** Chahat Singh, Pankaj Gharde, Harshal Tayade, Bhagyesh Sapkale

**Affiliations:** 1 General Surgery, Jawaharlal Nehru Medical College, Datta Meghe Institute of Higher Education and Research, Wardha, IND; 2 Medicine, Jawaharlal Nehru Medical College, Datta Meghe Institute of Higher Education and Research, Wardha, IND

**Keywords:** surgical considerations, individualized management, comprehensive assessment, timely intervention, differential diagnosis, surgical excision, subcutaneous mass, left lower abdomen, giant lipoma, lipoma

## Abstract

In the present case report, we discuss a case of an uncommon giant lipoma in the left lower abdomen of a 55-year-old female. The case is presented to highlight the need to consider lipoma as one of the possibilities in cases with subcutaneous masses. Despite its abnormal location and considerable magnitude, surgical excision under short general anesthesia resulted in successful outcomes without immediate complications. Finally, by comparing with similar circumstances, it is possible to recognize that individualized management strategies based on patient characteristics can benefit surgical considerations. The significant message is that timely intervention, comprehensive assessment, and teamwork are essential in achieving satisfactory outcomes among patients with rare cases of lipomas, such as this one.

## Introduction

Lipoma is a fatty mass usually located between the muscle layer underneath the skin [1]. Applying pressure makes subcutaneous lipomas readily migrate. However, lipomas can develop wherever fat cells are present; they are most frequently detected in the arms, back, shoulders, neck, chest, buttocks, and thighs [2]. A lipoma is removed surgically through a process known as lipoma excision [3]. It is usually done if the lipoma is proliferating or causing symptoms like pain or discomfort [1,3]. Lipomas come in various sizes, from tiny, pea-sized lumps to larger ones that can go up to several inches around [4]. Although a lipoma's size is not always related to its symptoms or consequences, more giant lipomas may be easier to see or more uncomfortable to feel, which increases the chance that it will need to be removed [5]. Regarding management, giant lipomas are classified as lesions more significant than 10 cm long or weighing more than 1,000 g, which poses unique challenges compared to smaller lipomas [6,7].

## Case presentation

**Figure 1:**
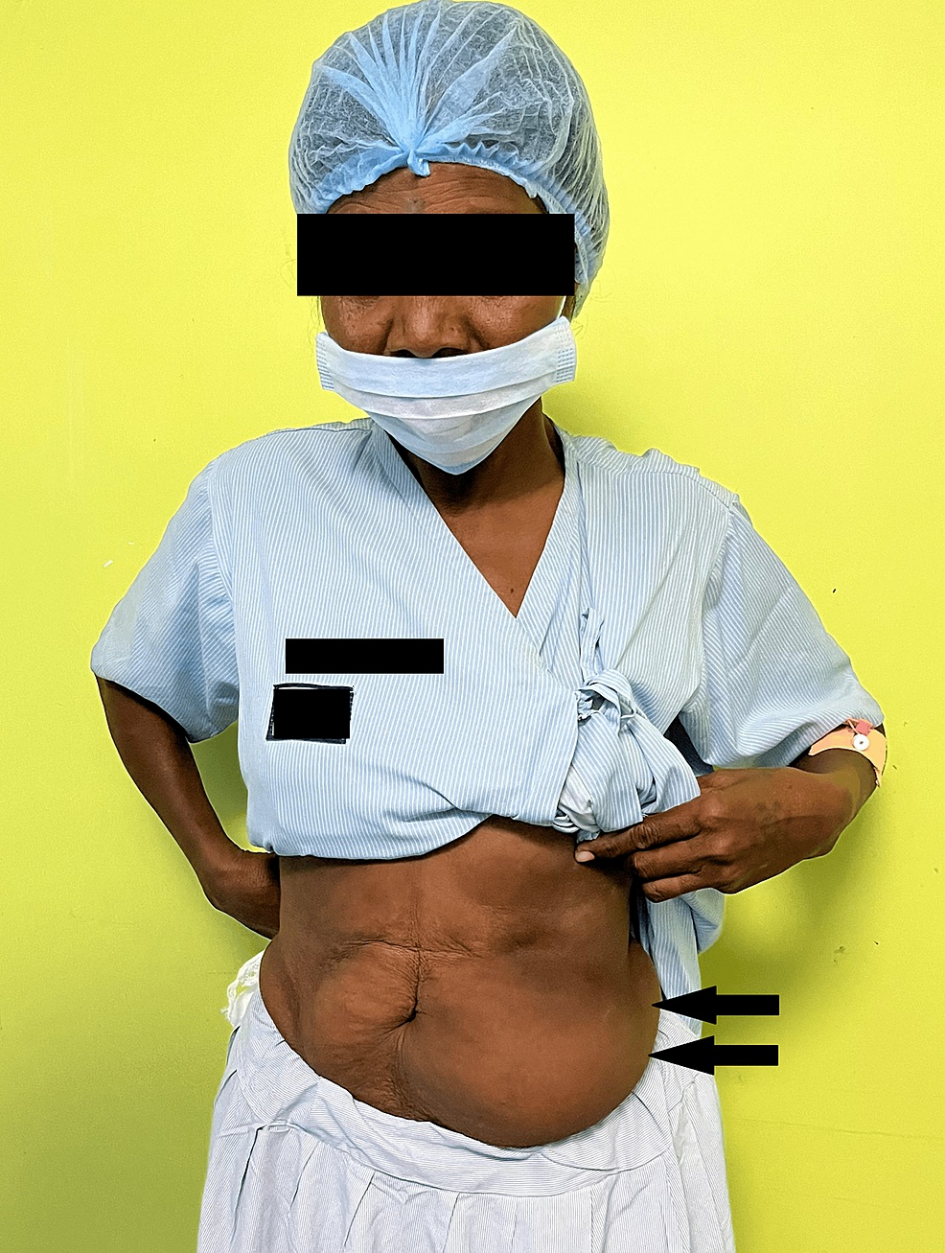
Swelling over the left lower abdomen.

A 55-year-old female presented to Acharya Vinoba Bhave Rural Hospital (AVBRH) with a chief complaint of swelling over the left lower abdomen, persisting for two years, initially noticed while taking a bath (Figure 1). She had no significant pain associated with the swelling but expressed concern about its progressive enlargement in size, prompting medical attention. The patient had no significant history of trauma or injury to the left lower abdomen. No relevant medical conditions, such as obesity or metabolic disorders, were noted, as they may contribute to the development or growth of lipomas. No significant family history of lipomas or other soft tissue tumors was present, which can provide insights into potential genetic predispositions.

**Figure 2:**
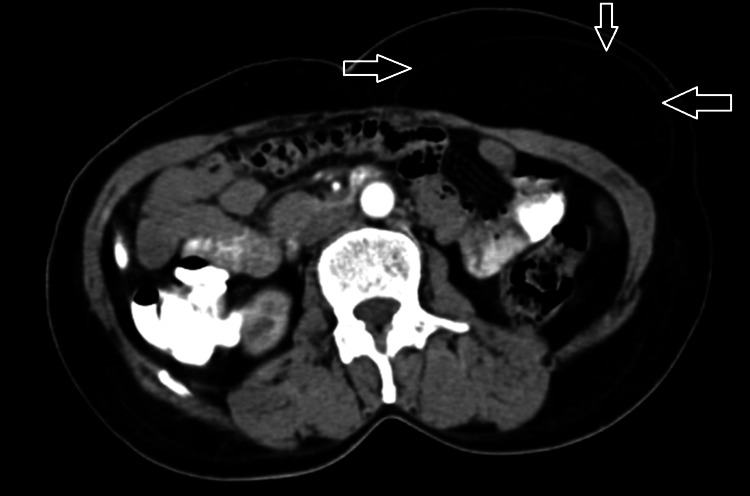
A computed tomography (CT) scan shows an encapsulated mass consistent with a lipoma, as indicated by arrows.

A soft, mobile mass was palpated in the subcutaneous tissue upon examination. Imaging Studies revealed a well-defined, encapsulated mass consistent with lipoma, albeit in an unusual location. Clinical investigations aimed to confirm the diagnosis of lipoma, assess the extent of the mass, and rule out any complications or associated conditions. Routine blood tests were assessed for the patient's overall health status, ruling out underlying medical conditions contributing to lipoma development. Fine-needle aspiration (FNA) was obtained from cells for microscopic examination, which confirmed the diagnosis of lipoma and ruled out other soft tissue tumors. Histopathological examination of excised tissue confirmed lipoma diagnosis and ensured complete removal. There were no signs of inflammation and skin changes overlying the swelling. A computed tomography (CT) scan shows an encapsulated mass consistent with a lipoma, as shown in Figure 2.

**Figure 3:**
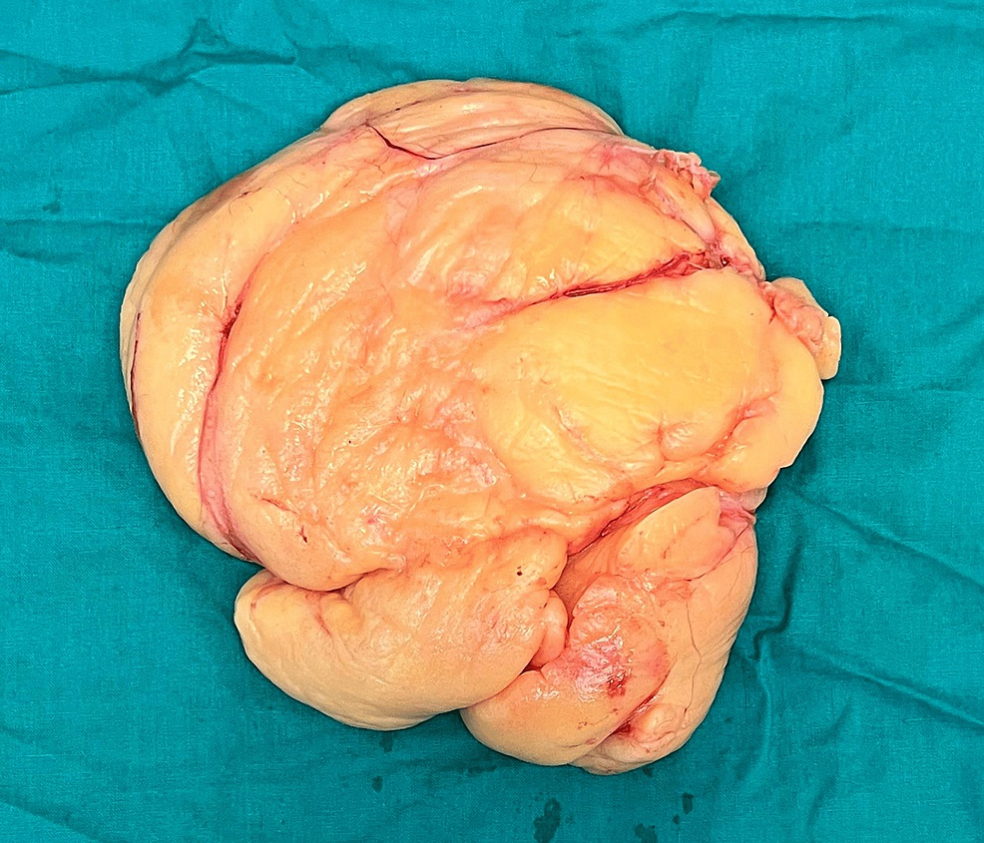
Lipoma after surgical excision.

Given the unique presentation and delayed diagnosis, surgical intervention was deemed necessary. The surgical team prepared the operating room and ensured all necessary instruments and equipment were readily available. A standard sterile technique was employed throughout the procedure to minimize the risk of infection. The patient underwent surgery under general anesthesia, during which a meticulous excision of the 13 cm giant lipoma was performed. The procedure involved a careful vertical incision using a sterile scalpel overlying the mass, followed by blunt dissection using a combination of dissecting scissors, forceps, and retractors to separate the lipoma from surrounding tissues. Hemostasis was achieved using electrocautery, ensuring minimal blood loss. The lipoma was then carefully dissected out in its entirety, preserving surrounding structures. Closure of the wound was performed in layers, with attention to achieving cosmetically pleasing results. The surgical team maintained clear communication and collaboration throughout the procedure, ensuring each step was performed with precision and attention to detail. Postoperative recovery was uneventful, with resolved swelling and no immediate complications noted. The patient was discharged with appropriate postoperative instructions and scheduled for follow-up to monitor for recurrence. This case underscores the importance of managing lipoma through surgical excision, even in atypical locations. This case is unique not only due to the atypical location of the lipoma but also its considerable size and the delayed presentation of the patient. Lipomas are commonly encountered benign soft tissue tumors, yet their occurrence in the left lower abdomen and attaining such dimensions are uncommon. A lipoma after surgical excision is shown in Figure 3.

The delayed diagnosis further adds to the case's complexity, emphasizing the importance of thorough evaluation and consideration of all differential diagnoses in patients presenting with subcutaneous masses. The successful management through surgical excision highlights the significance of timely intervention and comprehensive surgical care in achieving favorable outcomes, particularly in such exceptional cases.

## Discussion

Olafsdottir et al. described a 52-year-old female with a lipoma measuring 30 cm x 27 cm x 8.5 cm above the clavicle. Conversely, our case involved a 55-year-old female with a 13 cm giant lipoma located in the left lower abdomen [8]. These instances feature middle-aged women afflicted with substantial lipomatous tumors, albeit situated in distinct anatomical regions. While spinal anesthesia was attempted in the 52-year-old female's case, it proved unsuccessful due to the patient's weight, underscoring the complexities associated with managing surgical procedures in obese patients [8].

In the case of the 53-year-old male patient, as reported by Petca et al., a retroperitoneal tumor raised concerns about potential malignancy, prompting an exploratory laparotomy for excision [9]. The tumor spanned a considerable area from the colon's left splenic flexure to the lower abdomen. In contrast, our 55-year-old female patient's case of a giant lipoma limited to the lower left abdomen was simpler to diagnose than that of a retroperitoneal tumor [9]. Although the clinical presentation, use of diagnostic imaging, and need for surgical intervention were comparable in both cases, the patient demographics, tumor location, type, and surgical strategy differed, highlighting the need for tailored care methods in each case.

In the case of a 29-year-old female, as reported by Cha et al., with a mesenteric lipoma arising from the mesentery, displacing bowel loops and causing symptoms in the abdomen [10]. Both our 55-year-old female case and the 29-year-old female patient have abdominal discomfort and a mass, and diagnostic imaging is critical in determining the type and degree of the lesions [10]. The ages of the patients, as well as the precise locations and sizes of the lipomas, vary greatly, though. Surgical resection remains the primary recommendation for both patients, underscoring the importance of individualized management tailored to the patient's unique clinical characteristics and needs.

A 42-year-old female’s case was presented by Kher and Chakole with complaints of swelling on the left side of her back [11]. Our case and the case of a 42-year-old female both featured middle-aged female patients presenting with large, painless masses that grew gradually over several years. Diagnostic imaging techniques such as ultrasonography and CT scans were instrumental in confirming the lipomatous nature of the masses. Additionally, both cases underwent histopathological examination to confirm the benign nature of the lipomas. However, the location of the lipomas differs significantly, with the 42-year-old female’s case involving the back and our case describing an atypical occurrence in the left lower abdomen [11]. Furthermore, while both cases necessitated surgical excision, the specifics of the surgical approach and postoperative management differ based on the lipoma's location and size. Despite these disparities, the fundamental principles of diagnosis and management remain consistent, highlighting the importance of tailored approaches in addressing uncommon presentations of benign soft tissue tumors.

In the presented case of a 55-year-old female, differential diagnoses should be carefully considered to ensure accurate diagnosis and appropriate management. First, the possibility of liposarcoma, a malignant tumor arising from adipose tissue, must be evaluated due to its potential to mimic benign lipomas, especially given the size and location of the mass [1]. Additionally, hernias, such as inguinal or femoral hernias, should be ruled out as they can present as palpable masses in the lower abdomen and require imaging studies for accurate diagnosis [1,2]. Subcutaneous abscesses, secondary to infection, may also manifest as painful masses, necessitating clinical evaluation and imaging studies for differentiation from lipomas [1]. Furthermore, the differential diagnosis should consider benign conditions like dermatofibromas, epidermal inclusion cysts, and neurofibromas, each requiring specific diagnostic approaches such as biopsy or imaging for confirmation [4]. Finally, hematomas resulting from trauma or injury may present as tender masses in the subcutaneous tissue, necessitating thorough clinical history and imaging studies for differentiation from lipomas [1,4]. Differential diagnosis for a subcutaneous mass is depicted in Table 1.

**Table 1:** Differential diagnosis for a subcutaneous mass MRI, magnetic resonance imaging

Condition	Description	Key considerations	Diagnostic approach
Liposarcoma	Malignant tumor of fat tissue	- Mimics benign lipomas - Size and location may raise suspicion	Imaging studies, biopsy
Hernia (inguinal/femoral)	Internal organ protrudes through weak abdominal wall	- Palpable mass in the lower abdomen	Imaging studies (ultrasound and CT scan)
Subcutaneous Abscess	Pus-filled cavity due to infection	- Painful mass - redness, warmth may be present	Clinical evaluation, imaging studies (ultrasound)
Dermatofibroma	Noncancerous skin growth	- Firm, reddish-brown nodule	Biopsy
Epidermal inclusion cyst	Keratin-filled cyst	- Slow-growing, painless lump - may have central punctum	Clinical evaluation, aspiration
Neurofibroma	Noncancerous nerve sheath tumor	- May be painful or tender	Biopsy, imaging studies (MRI)
Hematoma	Blood collection due to trauma	- Tenderness may be present	Clinical evaluation, imaging studies (ultrasound)

Treatment options for giant lipomas, especially in uncommon locations like the left lower abdomen, are typically centered around surgical excision as the primary intervention [2]. However, alternative approaches may also be considered considering factors such as the patient's age, overall health status, and the size and location of the lipoma. These may include liposuction, a less invasive option for smaller, more accessible lipomas [1]. Additionally, observation could be warranted for asymptomatic cases, allowing regular monitoring to track any changes over time [2]. Minimally invasive techniques like endoscopic or laparoscopic excision may be suitable for certain cases, potentially leading to shorter recovery times [5]. Medical management options such as injectable medications have been explored, though their efficacy for giant lipomas is uncertain [1,3]. In cases where surgery is not feasible, radiation therapy might be considered a palliative option [6]. Furthermore, genetic counseling may be recommended for rare familial cases, providing insights into underlying genetic disorders and guiding treatment decisions [1]. Each option should be carefully evaluated based on the patient's clinical presentation and preferences, emphasizing a multidisciplinary approach for a tailored treatment plan. Treatment options for giant lipomas are presented in Table 2.

**Table 2:** Treatment options for giant lipomas.

Treatment option	Description	Advantages	Disadvantages	Considerations
Surgical excision	Traditional surgical removal of the lipoma	Widely used, effective for large lipomas	Scarring, longer recovery time	A standard approach for most giant lipomas
Liposuction	Fat suction technique for smaller, more accessible lipomas	Minimally invasive, smaller scar	May not be suitable for large lipomas, a higher recurrence rate	Option for smaller giant lipomas in appropriate locations
Observation	Monitoring the lipoma without intervention	Noninvasive, avoids risks of surgery	May cause anxiety, potential for delayed diagnosis of malignant tumors	Only for asymptomatic cases with close monitoring
Minimally invasive surgery (MIS)	Endoscopic or laparoscopic removal for specific cases	Shorter recovery time, less scarring	Requires specialized equipment and skills	Option for some giant lipomas depending on location and surgeon expertise
Injectable medications	Steroid injections to shrink the lipoma (limited research on giant lipomas)	Noninvasive, potentially reduces size	Limited effectiveness for giant lipomas, unknown long-term effects	May be considered for inoperable cases, but efficacy uncertain
Radiation therapy	Palliative treatment to manage symptoms like pain (not curative)	May provide pain relief	Potential side effects like tissue damage	Option for patients who cannot undergo surgery
Genetic counseling	For rare familial cases to understand underlying conditions	Provides insights for treatment decisions	Not applicable for most cases	Recommended for those with a family history of lipomas

## Conclusions

In conclusion, the case of the atypical giant lipoma in the left lower abdomen of a 55-year-old female underscores several significant points in the medical field. First, it highlights the importance of considering lipoma as a differential diagnosis for subcutaneous masses, even in uncommon locations. Second, it emphasizes successful management through timely surgical excision, showcasing the significance of comprehensive assessment and teamwork. Third, the case demonstrates the necessity of individualized management strategies based on patient characteristics for optimal outcomes. Overall, this case enhances our understanding of lipomas and reinforces the importance of tailored care in addressing rare and challenging medical conditions.

## References

[1] (2024). Lipoma: What Is It, Causes, Symptoms, Types, Treatment. https://my.clevelandclinic.org/health/diseases/15008-lipomas.

[2] Johnson CN, Ha AS, Chen E, Davidson D (2018). Lipomatous soft-tissue tumors. J Am Acad Orthop Surg.

[3] Salam GA (2002). Lipoma excision. Am Fam Physician.

[4] Lao QY, Sun M, Yu L, Wang J (2018). Lipofibromatosis: a clinicopathological analysis of eight cases. Zhonghua Bing Li Xue Za Zhi.

[5] Sanchez MR, Golomb FM, Moy JA, Potozkin JR (1993). Giant lipoma: case report and review of the literature. J Am Acad Dermatol.

[6] Drylewicz MR, Lubner MG, Pickhardt PJ, Menias CO, Mellnick VM (2019). Fatty masses of the abdomen and pelvis and their complications. Abdom Radiol (NY).

[7] Kosztyuova T, Shim TN (2017). Rapidly enlarging lipoma. BMJ Case Rep.

[8] Olafsdottir BE, Frodadottir HK, Runarsdottir R, Valsdottir EB (2018). Abdominal giant lipoma. Laeknabladid.

[9] Petca RC, Ambert V, Popescu RI, Mareş C, Petca AT, Berceanu C, Jinga V (2022). Half abdomen tumor - giant retroperitoneal lipoma: a case report and review of the literature. Rom J Morphol Embryol.

[10] Cha JM, Lee JI, Joo KR (2009). Giant mesenteric lipoma as an unusual cause of abdominal pain: a case report and a review of the literature. J Korean Med Sci.

[11] Kher C, Chakole S (2024). Giant lipoma: a case report. Cureus.

